# Radiomic Features Associated with Lymphoma Development in the Parotid Glands of Patients with Primary Sjögren’s Syndrome

**DOI:** 10.3390/cancers15051380

**Published:** 2023-02-22

**Authors:** Delia Doris Muntean, Lavinia Manuela Lenghel, Paul Andrei Ștefan, Daniela Fodor, Maria Bădărînză, Csaba Csutak, Sorin Marian Dudea, Georgeta Mihaela Rusu

**Affiliations:** 1 Radiology Department, Iuliu Hatieganu University of Medicine and Pharmacy, 400012 Cluj-Napoca, Romania; 2 Anatomy and Embryology, Morphological Sciences Department, Iuliu Hatieganu University of Medicine and Pharmacy, 400012 Cluj-Napoca, Romania; 3 Department of Biomedical Imaging and Image-Guided Therapy, Medical University of Vienna, General Hospital of Vienna (AKH), Waehringer Guertel 18-20, 1090 Vienna, Austria; 4 2nd Internal Medicine Department, Iuliu Hatieganu University of Medicine and Pharmacy, 400006 Cluj-Napoca, Romania

**Keywords:** radiomics, textural analysis, lymphoma, primary Sjögren’s syndrome, MRI

## Abstract

**Simple Summary:**

Patients diagnosed with primary Sjögren’s syndrome are characterized by an increased accumulation of mucosa-associated lymphoid tissue in the salivary and lacrimal glands due to chronic inflammation. Consequently, these patients present up to 40-fold higher risk of developing lymphoma, especially in the parotid gland, compared to the healthy population. Radiomics has recently proved its value in assessing tissue heterogeneity and proposing textural features that might become surrogates for biopsy. This retrospective study aimed to assess the potential value of radiomics in discovering textural analysis biomarkers associated with lymphoma development in the parotid glands of patients with primary Sjögren’s syndrome based on MR images, which might provide new directions in assessing the disease.

**Abstract:**

Non-Hodgkin Lymphoma (NHL) represents a severe complication and the main cause of morbidity in patients with primary Sjögren’s syndrome (pSS). This study aimed to assess the role of textural analysis (TA) in revealing lymphoma-associated imaging parameters in the parotid gland (PG) parenchyma of patients with pSS. This retrospective study included a total of 36 patients (54.93 ± 13.34 years old; 91.6% females) diagnosed with pSS according to the American College of Rheumatology and the European League Against Rheumatism criteria (24 subjects with pSS and no lymphomatous proliferation; 12 subjects with pSS and NHL development in the PG, confirmed by the histopathological analysis). All subjects underwent MR scanning between January 2018 and October 2022. The coronal STIR PROPELLER sequence was employed to segment PG and perform TA using the MaZda5 software. A total of 65 PGs underwent segmentation and texture feature extraction (48 PGs were included in the pSS control group, and 17 PGs were included in the pSS NHL group). Following parameter reduction techniques, univariate analysis, multivariate regression, and receiver operating characteristics (ROC) analysis, the following TA parameters proved to be independently associated with NHL development in pSS: CH4S6_Sum_Variance and CV4S6_Inverse_Difference_Moment, with an area under ROC of 0.800 and 0.875, respectively. The radiomic model (resulting by combining the two previously independent TA features), presented 94.12% sensitivity and 85.42% specificity in differentiating between the two studied groups, reaching the highest area under ROC of 0.931 for the chosen cutoff value of 1.556. This study suggests the potential role of radiomics in revealing new imaging biomarkers that might serve as useful predictors for lymphoma development in patients with pSS. Further research on multicentric cohorts is warranted to confirm the obtained results and the added benefit of TA in risk stratification for patients with pSS.

## 1. Introduction

Primary Sjögren’s syndrome (pSS) represents an inflammatory autoimmune disease, commonly affecting the salivary and lacrimal glands, resulting in the progressive destruction of the glandular parenchyma, which is replaced by fat and fibrous tissue. Consequently, patients with pSS suffer from exocrine dysfunction and sicca syndrome [1,2].

The hallmark of the pathological process in patients with pSS is the accumulation of mucosa-associated lymphoid tissue (MALT) due to chronic inflammation [3]. This tissue represents a substrate for non-Hodgkin lymphoma (NHL) development in this group of patients (especially the NHL-MALT histologic subtype). It is estimated that patients with pSS have an increased risk of developing lymphoma, up to 40-fold higher than in the general population [4]. Therefore, predicting the pSS outcome both at disease onset and during follow-up is of paramount importance.

Clinical and biological predictors of lymphoma in pSS have been assessed, the two major ones being persistent salivary gland swelling and cryoglobulinemic vasculitis. Composite indexes/scores have also been proposed as valuable tools in predicting lymphoma [5,6]. Several imaging features proved to be associated with lymphoma development in pSS, such as the severity of main salivary gland (MSG) parenchymal destruction based on the ultrasonographic aspect [7,8], a parotid gland (PG) stiffness value > 11.5 kPA assessed using 2D shear-wave elastography [9], and an increased diffusion restriction with low apparent diffusion coefficient [10]. However, despite the obtained results, biopsy and histopathological analysis remain mandatory for lymphoma diagnosis [11].

Recently, radiomics has emerged as a rapidly evolving post-processing imaging technique that can extract high-dimensional quantitative data from radiological images and aims to surpass the limits of the subjective observational imaging assessment [12,13]. Textural analysis (TA) features proved to be correlated to tissue heterogeneity at a cellular level [14,15] and, therefore, might represent novel biomarkers that could improve diagnostic accuracy and facilitate the decision-making process for clinicians, especially considering the current age of targeted treatment and patient-customized medicine [16,17].

In the field of salivary gland imaging, the role of radiomics was mainly assessed in the following two clinical settings: oncology and radiation-induced xerostomia, while few studies focused on inflammatory pathologies [18].

Radiomic studies with a specific focus on pSS are scarce. One study proposed a radiomic-based evaluation of the pSS scoring system using MSG ultrasound images and identified the best-performing classifier (multilayer perceptron) among the considered artificial intelligence algorithms [19]. Additionally, the efficiency of deep learning algorithms was tested for the automated PG segmentation on ultrasound images [20].

Regarding the role of radiomics in pSS using MRI, one study revealed textural features in the lacrimal gland’s parenchyma that were able to distinguish between patients with pSS and healthy controls [21]. Another MRI radiomic study found imaging biomarkers that could stage disease activity in pSS. The histogram derived from the PG segmentation on the ADC map provided quantitative parameters that reflected different tissue characteristics between pSS patients and healthy volunteers [22]. Although MRI is a useful technique in the local staging of malignant PG tumors and pSS-associated lymphomas of salivary and lacrimal glands [23], to the best of our knowledge, no radiomic study regarding lymphomatous proliferation in pSS has been performed so far.

NHL represents the main complication and cause of morbidity in pSS patients, and the gold standard diagnostic method remains biopsy, which is an invasive procedure with a nonnegligible risk of sampling error [23,24]. Although pSS-associated lymphomas often present an indolent evolution, these malignancies still present the risk of dissemination to other mucosal sites or organs [25].

Therefore, the aim of this radiomic study was to identify alternative, non-invasive MRI textural features in the PG parenchyma associated with NHL development in patients with pSS that might serve as prognostic biomarkers, complement biopsy, and provide new directions in assessing the disease.

## 2. Materials and Methods

This study was performed according to the Declaration of Helsinki and received approval from the Ethical Committee of the “Iuliu Hațieganu” University of Medicine and Pharmacy Cluj-Napoca (registration number: 43; date: 11 February 2022). Due to the study’s retrospective design, all participants’ informed consent was waived.

### 2.1. Patients and Standard Reference

This retrospective nonrandomized study was conducted on patients with previously documented pSS who underwent head and neck MRI examination between January 2018 and October 2022 to assess MSG.

The inclusion criteria were the fulfillment of the American College of Rheumatology and the European League Against Rheumatism (ACR/EULAR) diagnostic criteria (2016) for pSS [26] and age older than 18 years. One rheumatologist with 5 years of clinical experience evaluated all patients. The Schirmer’s test and the unstimulated whole salivary flow (UWSF) were assessed for all subjects, and the EULAR Sjögren’s Syndrome disease activity index (ESSDAI) was computed [27]. Biological analysis (anti-Ro/La autoantibodies and rheumatoid factor (RF)) was performed for each patient.

The exclusion criteria were secondary Sjögren’s syndrome (in patients with systemic lupus erythematosus, rheumatoid arthritis, progressive systemic sclerosis, or mixed connective tissue disease), sialolithiasis, previous neck radiation, history of hepatic virus C infection and patients with sicca syndrome that did not fulfill the pSS criteria.

After applying the inclusion and exclusion criteria, a cohort of 36 consecutive patients was constituted.

For the statistical analysis of the clinical and biological features, subjects were divided into two groups: one included 12 patients with pSS and NHL at the time of the MRI examination (pSS NHL group), and another control group of 24 pSS patients without lymphomatous transformation (pSS control group).

The gold standard for pSS patients with NHL was the histopathological result obtained after core needle biopsy or surgery. All samples were analyzed in the same institution. All subjects in the pSS NHL group were confirmed with NHL-MALT subtype and on MRI presented solitary solid nodules or masses identified due to the increased diffusion restriction and extremely low apparent diffusion coefficients (<0.650).

Patients in the control group were followed-up at 6 months and 1 year after the MRI exam. They did not present any clinical or imaging changes (assessed with the ultrasonography of MSG) suggesting lymphomatous transformation compared to the initial evaluation.

For the radiomic analysis, a total of 65 PGs were included (representing the training dataset). PGs were divided into two groups: 48 PGs were included in the pSS control group (both PGs of one patient underwent segmentation and feature extraction), while 17 PGs were included in the pSS NHL group (seven patients with unilateral involvement, and five patients with bilateral involvement, respectively).

### 2.2. Image Acquisition

All patients underwent head and neck MRI exams on a 1.5 Tesla scanner (SIGNA™ Explorer, General Electric) using an eight-channel high-resolution head coil. The MRI protocol comprised the following standard sequences: axial and coronal FSE T1-WI and FSE T2-WI, coronal STIR PROPELLER (short tau inversion recovery with periodically rotated overlapping parallel lines with enhanced reconstruction), coronal SE T1-WI with fat saturation obtained after intravenous administration of gadolinium chelate, diffusion-weighted imaging with the ADC map, and heavily three-dimensional T2-WI (MRI sialography) covering all MSG.

For the TA, the coronal STIR PROPELLER sequence was used, acquired employing the following specifications: field of view 256 × 256 mm; slice thickness 3 mm; slice gap 0.3 mm; echo time 60 ms; repetition time 3500 ms; inversion time 1900 ms; bandwidth 437.1 Hz/pixel; acquisition time 4 min.

### 2.3. Texture Analysis Protocol and Statistical Analysis

#### 2.3.1. Image Preprocessing and Segmentation

One head and neck specialized radiologist reviewed each MRI examination on a dedicated workstation (General Electric, Advantage workstation, 4.7 edition) and identified the best artifact-free slice for assessing PG on the STIR PROPELLER sequence. After anonymization, the selected images were retrieved in a DICOM format and imported into an open-source texture analysis software, MaZda 5 (Institute of Electronics, Technical University of Lodz, Lodz, Poland) [28].

To decrease image variations in brightness and contrast that might impair the natural texture of PG, the first step in the MaZda program was to apply a grey-level normalization technique to all images. The mean (µ) and standard deviation (σ) of grey levels of voxels inside the ROIs were computed, and all outlier voxels (beyond µ ± 3σ) were consequently removed. A preprocessing wavelet filter was applied, using high and low bandpass filters.

A total of 65 PGs divided into two groups (48 PGs in the pSS control group and 17 in the pSS NHL group) underwent segmentation and feature extraction.

**Figure 1:**
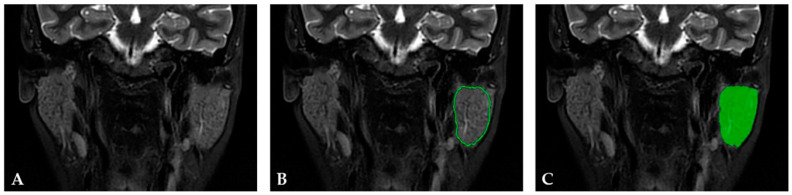
(**A**) MRI coronal STIR PROPELLER sequence of a 57-year-old female patient diagnosed with primary Sjögren’s syndrome. (**B**) The region of interest (ROI) covering almost the entire parotid gland was semiautomatically delineated by the software following geometry and gradient coordinates (green circle). (**C**) The final ROI after the manual correction was performed (green area).

The segmentation process for the pSS control group implied incorporating each PG into a 2D region of interest (ROI) using a semiautomatic algorithm. Firstly, a seed was placed inside the PG parenchyma, and the ROI was automatically traced following the PG contour using gradient and geometrical coordinates. When necessary, manual corrections were further applied. An example of PG segmentation is shown in Figure 1. The segmentation was performed for both PGs of one subject.

**Figure 2:**
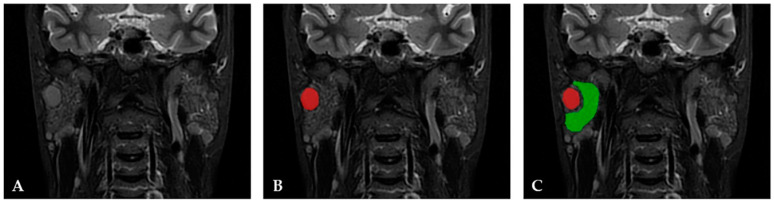
(**A**) MRI coronal STIR PROPELLER sequence of a 45-year-old female patient diagnosed with primary Sjögren’s syndrome and non-Hodgkin lymphoma, MALT subtype in the right parotid gland. (**B**) The software automatically traced the region of interest (ROI) covering the focal lesion corresponding to lymphoma using geometric and gradient coordinates (red area). (**C**) A second ROI was manually delineated (green area), covering the parotid gland’s parenchyma surrounding the focal lesion (this 2D segmentation was used for the textural analysis).

The segmentation process for the pSS NHL group was performed first by automatically tracing a 2D ROI that comprised the focal lesion corresponding to NHL using gradient and geometrical coordinates. Then, the PG parenchyma outside the lesion was manually delineated using a nonoverlapping 2D ROI at a 2 mm distance from the first 2D ROI (Figure 2).

The 2D ROIs represented the regions in which the radiomic features were calculated.

#### 2.3.2. Feature Extraction

From each segmented ROI, MaZda software automatically extracted a total of 275 parameters belonging to six texture classes (Absolute gradient, Histogram, Co-occurrence Matrix, Run Length Matrix, Auto-regressive Model, and Wavelet transformation). Details regarding the extracted radiomic features and the computation settings of each class are shown in Table 1.

#### 2.3.3. Feature Selection and Statistical Analysis

From the total of 275 extracted features, the MaZda program allowed the selection of the most discriminative features through several preset reduction techniques. As a first step, the probability of classification error and average correlation coefficients (POE + ACC) reduction technique was applied [29,30], and a set of 10 features was generated. This algorithm, available within the MaZda program, selects features with the highest discriminative ability while being poorly correlated, thus making them suitable for building prediction models.

To assess the stability and reproducibility of the selected TA feature set after computing the POE + ACC reduction technique, 30 PGs (randomly chosen from both studied groups) underwent re-segmentation 1 month apart from the initial procedure. The same radiologist redefined another ROI and a second round of feature extraction was performed. Then, the intraobserver reproducibility of the radiomic features was assessed using the intraclass correlation coefficient (ICC).

Radiomic parameters that presented an ICC higher than 0.850 were regarded as stable, and their corresponding absolute values from the initial segmentation were considered suitable for the subsequent statistical analysis.

A univariate analysis test (Mann–Whitney U) was further performed to assess which features were best suited to discriminate between the pSS control group and the pSS-NHL group. The statistically significant level was set at a *p*-value lower than 0.05. All texture parameters that showed univariate analysis results above this threshold were excluded from further processing. The receiver operating characteristic (ROC) analysis was performed, with the calculation of the area under the curve (AUC) for parameters that demonstrated statistically significant results in the univariate analysis (*p* < 0.05). Optimal cutoff values were chosen using a common optimization step that maximized the Youden index. Sensitivity (Se), specificity (Sp), positive likelihood ratio (+LR), and negative likelihood ratio (−LR), with their corresponding 95% confidence intervals (CI), were computed from the same data without further adjustments.

Parameters that showed statistically significant results in the univariate and ROC analysis were included in a multiple regression using the “enter” input model. The resulting features independently associated with lymphoma development in patients with pSS were used to generate a radiomic model, computed using the regression coefficients.

**Figure 3:**
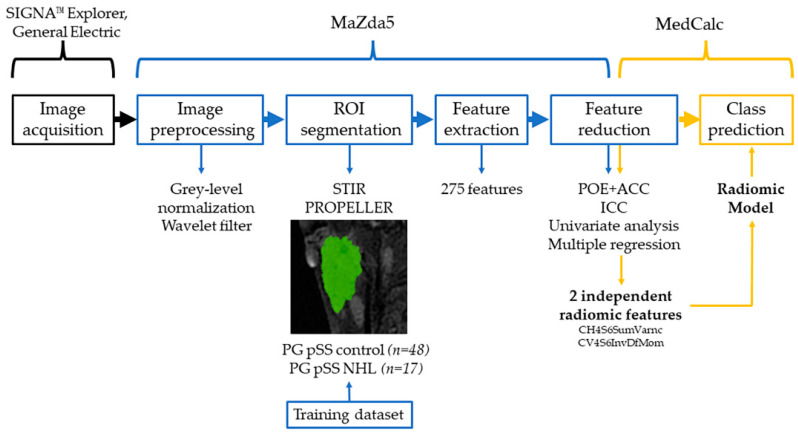
Radiomics workflow diagram. ROI = region of interest; STIR PROPELLER = short tau inversion recovery with periodically rotated overlapping parallel lines with enhanced reconstruction; PG = parotid gland; pSS = primary Sjögren’s syndrome; NHL = non-Hodgkin lymphoma; POE + ACC = probability of classification error and average correlation coefficients; ICC = Intraclass Correlation Coefficient, SumVarnc = Sum Variance; InvDfMom = Inverse Difference Moment.

An overview of the radiomic workflow used in this study is offered in Figure 3.

This step-by-step feature selection method was used in previous texture analysis studies [31,32], and the resulting parameters demonstrated good discriminative ability.

The statistical analysis regarding the clinical and biological features implied assessing the differences between the means or medians using the independent-samples T test or Mann–Whitney U test, as necessary. The exact Fisher test was used to evaluate the association between categorical variables.

The statistical analysis was performed using the commercially available dedicated software, MedCalc version 14.8.1 (MedCalc Software, Mariakerke, Belgium).

## 3. Results

A total of 36 patients diagnosed with pSS referred to our imaging department during the study period (mean age 54.93 ± 13.34; age range 29–83) were included in this study (Table 2).

Statistically significant risk factors for NHL development in our cohort of patients with pSS were the ESSDAI score value and the positive presence of the rheumatoid factor. Subjects in the pSS NHL group presented a higher disease activity than the pSS control group, using an ESSDAI score cutoff value of 5 (*p* < 0.001). The overall ESSDAI score values were higher in the pSS NHL group (*p* < 0.001). Disease duration did not influence the lymphomatous transformation (*p* > 0.05). The rheumatoid factor was present in all patients in the pSS NHL group and only 62.5% of the subjects in the pSS control group (*p* = 0.016).

For the textural analysis, 48 PGs from the pSS control group and 17 PGs from the pSS NHL group underwent segmentation, and a total of 275 radiomic features were extracted. Following the POE+ACC reduction technique, 10 unique texture features with the highest discriminatory values between the two studied groups were selected. Seven of the 10 previously selected texture features showed statistically significant results in the univariate analysis (Table 3). All selected parameters presented high ICC values (≥0.850). One variation of Sum Variance, Run Length NonUniformity, and Sum Average were excluded from further analysis, as the *p*-value exceeded 0.05.

The receiver operating characteristics (ROC) analysis was further performed. Three parameters (CH4S6SumVarnc, CV4S6InvDfMom, and Perc1) presented 88.24% sensitivity in differentiating between the two groups of patients, with specificities of 64.58%, 77.08%, and 62.50%, respectively. The highest area under the curve (AUC) was reached by CV4S6InvDfMom (0.875). The extended ROC analysis results are presented in Table 4.

The seven texture features that showed statistically significant results in the univariate analysis, high ICC values, and ROC analysis were included in the multivariate regression.

The multivariate analysis showed a coefficient of determination (R^2^) of 0.5524, an adjusted R^2^ of 0.4975, a multiple correlation coefficient of 0.7433, and a residual standard deviation of 0.3140 (Table 5). Four parameters proved to be independent predictors for NHL development in patients with pSS (CH4S6SumVarnc, Perc90, Mean, CV4S6InvDfMom). A radiomic model (RM) was generated, including two independent parameters (CH4S6SumVarnc and CV4S6InvDfMom) revealed in the multivariate analysis. Two parameters (Mean, Perc90) were excluded from the model due to the high variance inflation factor (VIF), indicating multicollinearity. RM was computed using the regression coefficients (RM = −27.7065 + 0.00417CH4S6SumVarnc + 3.3534CV4S6InvDfMom).

At the cutoff value ≥ 1.556, the RM was associated with NHL development with high sensitivity and specificity (94.12% and 85.42%, respectively), presenting an AUC of 0.931. (Table 6).

**Figure 4:**
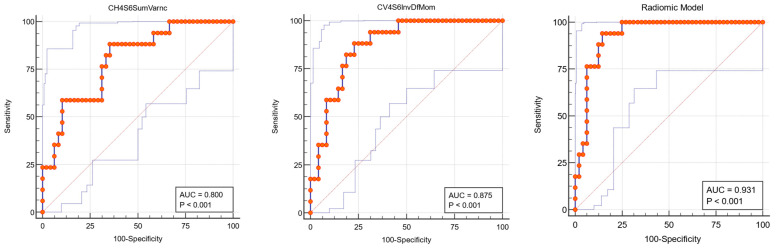
Receiver operating characteristics (ROC) curve of the independent parotid glands’ texture features (CH4S6SumVarnc, CV4S5InvDfMom) and the resulting radiomic model, associated with lymphoma development in patients with primary Sjögren’s syndrome (orange dotted line—ROC curve; thin blue lines—ROC Confidence Interval).

The areas under the ROC curve of the independent features associated with lymphoma development in pSS and the resulting radiomic model are depicted in Figure 4.

## 4. Discussion

The exocrine glands of patients with pSS are characterized by an increased content of mucosa-associated lymphoid tissue, which consequently increases the risk of developing lymphoma [3].

The ability to forecast the pSS outcome on disease onset and during follow-ups is still limited despite years of research. Significant clinical and biological predictors of lymphoma in pSS proved to be persistent enlargement of the MSG (defined as lasting at least 2 months) and mixed cryoglobulinemia [3,5,33].

Currently, there are no studies to certify the role of any imaging technique as a validated predictive tool in pSS. Therefore, discovering noninvasive imaging biomarkers that might be associated with lymphoma development in pSS represents a crucial step for further clinical and applied research.

MRI represents a valuable imaging technique in pSS diagnosis, allowing both the anatomical assessment of MSG by using T1- and fat-suppressed T2-weighted images and the functional evaluation with MRI sialography, based on the spontaneously increased signal of stagnant fluids on heavily T2-weighted sequence and simultaneously signal suppression of the adjacent tissue [2]. The association of more than 5% fat areas with diminished intact parenchyma replaced by areas with increased signal in the MSG on T2-weighted images with fat saturation, together with an increased number of foci of salivary duct ectasia (≥6), reached a 96.4% sensitivity and 100% specificity for pSS diagnosis [34]. MRI sialography of PG has outstanding diagnostic performance in pSS (sensitivity and specificity ranging between 83–96% and 83–100%) [34,35,36]. MRI also proves helpful in parotid lymphoma diagnostic guidance and local staging [37,38]. According to the algorithm proposed by Jousse-Joulin et al. [11], the association of solid and cystic lesions with very low apparent diffusion coefficient values requires a biopsy, as there is high lymphoma suspicion.

Recently, radiomics proved to be a promising tool in oncological imaging, especially in diagnosing cancer, evaluating the response to therapy, or predicting prognosis [16,17].

Therefore, the present radiomic study aimed to assess the role of TA in discovering imaging biomarkers in the PG of patients with pSS that are associated with lymphoma development and could potentially become surrogates of the histopathological results.

Using fat saturation techniques, the PG parenchymal architecture was outlined by suppressing additional signals originating from interlobular fat structures [39,40]. Therefore, the STIR sequence was selected to perform PG segmentation and TA feature extraction. The STIR technique provided a more uniform fat suppression than the fat-saturated FSE technique, especially for cervical MR imaging, where the air-filled structures and complex anatomy might generate susceptibility effects and cause magnetic field inhomogeneity [41]. Moreover, the PROPELLER technique was utilized to reduce motion artifacts, improve image quality and obtain a more homogenous set of images [42].

**Figure 5:**
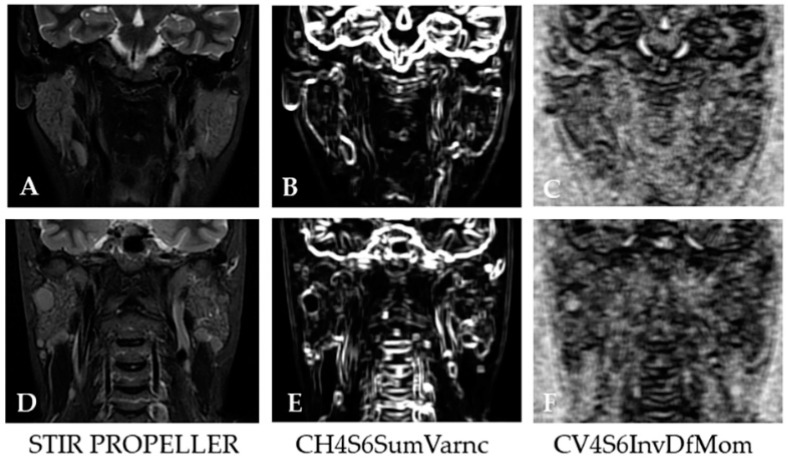
Texture maps presenting the distribution of the independent features (CH4S6SumVarnc, CV4S6InvDfMom) associated with NHL development in PG of patients with pSS on the selected MRI coronal STIR PROPELLER image. (**A**–**C**) images of a patient with pSS and without lymphomatous proliferation in the parotid gland; (**D**–**F**) images of a patient with pSS and NHL development in the right parotid gland.

In this study, two independent texture analysis features were found to be associated with lymphoproliferation in pSS (Figure 5): CH4S6SumVarnc and CV4S6InvDfMom, each presenting a good AUC (>0.750).

The combination radiomic model proved to perform better than the individual parameters, reaching an AUC of 0.931.

Sum variance (CH4S6SumVarnc) and inverse difference moment (CV4S6InvDfMom) were obtained from the co-occurrence matrix. Within a given ROI, sum variance measures the deflection extent of the sum of grey-level intensity distribution from the mean grey-level value [43]. Therefore, sum variance is a parameter that reflects heterogeneity by emphasizing the deviation of neighboring grey levels from the mean in the co-occurrence matrix [44]. In our study, sum variance presented higher values in the pSS NHL group, reflecting more inhomogeneous parenchyma of the PG in comparison to the pSS control group (234.60 vs. 199.24, *p* < 0.001).

The inverse difference is an indicator of homogeneity [45]. A wide range in levels of grey-level co-occurrences is less quantified and consequently lowers the overall feature’s value. In other words, the maximum value for this feature is obtained if there is no difference in the grey levels. The inverse difference moment is conceptually similar to the inverse difference feature. However, it gives less weight to items further away from the diagonal and is also linked to homogeneity [46,47]. In the pSS NHL group, CV4S6InvDfMom proved to be lower than in the pSS control group (0.10 vs. 0.18, *p* < 0.0001); therefore, a large grey level variation equivalent to an increased parenchymal inhomogeneity of PG favors NHL development.

Our results show that a high PG parenchymal heterogeneity quantified by TA features is associated with NHL development. This observation agrees with other studies that proved that severe parenchymal MSG destruction, with a consequently increased inhomogeneity assessed on ultrasonography, could be a risk factor for progression to MALT lymphoma [7,8,9].

To the best of our knowledge, this is the first study to assess the role of radiomics in depicting features associated with lymphoma development in the PG of patients with pSS, and the obtained preliminary results are promising. However, this study has several important limitations that need to be addressed.

Firstly, TA was performed in a relatively small sample of PG (*n* = 65) that represented the training dataset. Consequently, the small training dataset might lead to unintentional overfitting, which would hinder the generalization of the radiomic model. To address this issue, a validation dataset is warranted [48,49]. Generally, approximately 70% of the acquired dataset is used for training, and the remaining samples are used to evaluate the classifier’s performance on another validation dataset [50]. We were unable to split the acquired data into a training and validation dataset, due to the limited number of observations in the pSS-NHL group (*n* = 17, 26% of all observations). Although pSS is one of the most common autoimmune disorders, its prevalence in the general population is still relatively low (0.06% worldwide in the general population) [51]. This fact, together with the monocentric aspect of this study, has unfortunately contributed to a limited number of patients with pSS referred to our imaging center, and an even lower number of pSS patients that developed lymphoma.

Therefore, the discovered radiomic features associated with lymphoma development in the parotid glands of patients with pSS could not be tested on a separate validation set in this study. This would have significantly increased the reliability of the obtained results and counteracted any potential overfitting. More extensive multicentric studies (which would also provide external datasets) are required to collect a sufficient number of pSS subjects that could be appropriately used for training and validation groups in AI algorithms based on TA.

Moreover, important limitations in MRI radiomic studies are related to the presence of confounding factors, some related to differences in image acquisition [52,53,54,55], scanner [56], or vendor [57,58] differences. Variations in image acquisition settings such as technical parameters (matrix size, time of repetition, time of echo, signal-to-noise ratio,) or voxel size (slice thickness, pixel size) may lead to pictures of varying quality, which may impact the performance of the radiomic signatures and limits generalization [53,55].

MRI radiomic studies also present great potential in identifying predictive biomarkers in several head–neck pathology studies. However, due to the high variability in methodology, collective and accurate data assessment is limited [58].

Radiomics reporting guidelines, including Radiomics Quality Score (RQS) [59] or the Image Biomarker Standardization Initiative (IBSI) [60], proposed different approaches to conduct reproducible and generalizable radiomics studies. However, there is still a lack of consensus on how to control and reduce the effect of potential confounding factors. RQS stresses the importance of reporting exact details of the used imaging protocol, but no reliable strategies for reducing the confounding effects have been provided. Conversely, the IBSI guideline emphasizes having a pre-processing standardized algorithm for feature extraction and focuses less on limiting the confounding factors. Some studies have assessed radiomic feature robustness by using test-retest repeated scans or multiple MRI scans [61,62].

The means to control confounding factors in our study were by using a standardized MRI protocol, with fixed technical parameters for all patients with pSS that were examined in our department, and by performing image processing and computation before feature extraction. Although we strongly acknowledge the importance of feature robustness assessment that impacts model generalization [55], due to the retrospective nature of this study, additional experiments could not be performed, and therefore, we could not test the effect of confounding parameters. This represents an important limitation of the present study but represents an important objective for future prospective radiomic studies in our department.

Another limitation of the study is that the radiomic features were extracted from a 2D ROI segmentation. However, in daily clinical practice, a 3D segmentation of the PG, which has an irregular shape, might be harder to adopt, given the longer time required for segmentation and possible increased operator variability.

Moreover, differences between PGs with lymphoma and the contralateral PGs without lymphoma in the pSS NHL group could not be tested, given the low number of subjects with unilateral PG lymphomatous proliferation. Finally, since all patients in the pSS NHL group were diagnosed with MALT lymphoma, one cannot draw generalizations about other NHL subtypes.

## 5. Conclusions

This study suggests that radiomic analysis of the parotid gland’s parenchyma performed on MR images has the potential to reveal new imaging biomarkers that reflect tissue heterogeneity associated with lymphoma development in patients with pSS. However, the results obtained in this study must be confirmed in larger prospective studies, using ideally multicentric cohorts, to validate the role of textural analysis in the risk stratification of patients with pSS.

## Figures and Tables

**Table 1:** The extracted radiomics features and the computation settings of each category.

Category	Feature	Computation	Variation	Number of Features
Histogram	Mean, Kurtosis, Percentile 01/10/50/90/99% Skewness, Variance	-	-	9
Co-occurrence matrix	Angular second moment, Contrast, Correlation, Difference entropy, Difference Variance, Entropy, Inverse difference moment, Sum average, Sum entropy, Sum of squares, Sum variance	6 bits/pixel	Computed 20 times for distance values from 1 to 5	220
Run length matrix	Fraction of image in runs, Grey level nonuniformity, Long run emphasis, Run length nonuniformity, Short run emphasis	6 bits/pixel	Computed four times for horizontal, vertical, 45°, and 135° directions	20
Gradient	Kurtosis, Mean, Percentage of pixels with nonzero gradient, Skewness, Variance	4 bits/pixel	-	5
Autoregressive model	Sigma, Teta 1–4	-	-	5
Wavelet	Wavelet energy with high- and low-pass filters	8 bits/pixel	4 scales	16

**Table 2:** Patient clinicobiological features.

Feature	All Patients (*n* = 36)	pSS Control Group (*n* = 24)	pSS NHL Group (*n* = 12)	*p*
Age (years)	54.93 ± 13.34	58.79 ± 12.44	46.92 ± 12.50	0.013
Gender (female)	33 (91.6)	23 (95.8)	10 (83.3)	0.207
BMI (kg/m^2^)	26.11 ± 4.39	25.23 ± 3.98	27.39 ± 5.01	0.130
Disease duration (months)	34 [17, 50]	29 [11, 46]	37 [20, 69]	0.416
Disease duration				0.349
<5 years	24 (66.7)	21 (87.5)	3 (25)	
≥5 years	12 (33.3)	3 (12.5)	9 (75)	
ESSDAI score	2 [0, 9]	0 [0, 2]	13 [9, 15]	<0.001
Disease activity				<0.001
Low (ESSDAI < 5)	22 (61.1)	20 (83.3)	2 (16.6)	
Moderate-high (ESSDAI ≥ 5)	14 (38.9)	4 (16.7)	10 (83.4)	
Positive Schirmer’s test	33 (91.6)	21 (87.5)	12 (100)	0.522
UWSF (mL)	1.24 ± 0.34	1.23 ± 0.34	1.25 ± 0.28	0.861
Anti-Ro/La autoantibodies	32 (88.9)	20 (83.3)	12 (100)	0.139
Rheumatoid factor	27 (75)	15 (62.5)	12 (100)	0.016

The results are expressed as mean ± standard deviation, median and [interquartile range], or percentage (%), *n* = number of patients, BMI = body mass index, ESSDAI = EULAR Sjögren’s syndrome disease activity index; UWSF = unstimulated whole salivary flow.

**Table 3:** Texture features associated with lymphoma development in patients with pSS after the POE + ACC (probability of classification error and average correlation coefficients) reduction technique and the univariate analysis results.

Texture Parameters	PG pSS Control Group (*n* = 48)		PG pSS NHL Group (*n* = 17)		*p*	ICC
	Median	IQR	Median	IQR		
CH4S6SumVarnc	199.24	175.39–221.77	234.60	214.35–257.19	0.0004	0.956
WavEnHL_s-4	270.38	186.15–370.19	497.10	283.74–727.07	0.0094	0.910
Perc90	33,280.50	33,186.00–33,354.50	33,512.00	33,359.75–33,563.50	0.0001	0.922
Mean	33,147.03	33,101.45–33,229.68	33,377.18	33,230.06–33,389.89	0.0001	0.933
CV4S6InvDfMom	0.18	0.16–0.22	0.10	0.10–0.13	<0.0001	0.924
CH3S6Correlat	0.14	0.04–0.25	0.405	0.17–0.49	0.0027	0.901
CN1S6SumVarnc	349.22	327.72–369.30	360.04	320.52–388.26	0.3547	0.897
RNS6RLNonUni	1371.17	1104.56–1720.21	1603.50	1272.27–2083.16	0.2383	0.905
Perc1	32,990.50	32,942.00–33,050.00	33,109.00	33,028.25–33,157.25	0.0005	0.911
CV5S6SumAverg	65.00	64.50–65.29	62.86	59.58 to 65.09	0.4737	0.934

IQR = interquartile range; *p* = statistical significance level; ICC = intraclass correlation coefficient; SumVarnc = Sum Variance; WavEnHL = Wavelet Energy High-Level; Perc = percentile; InvDfMom = inverse difference moment; Correlat =correlation; RLNonUni = Run Length NonUniformity; SumAverg = Sum Average.

**Table 4:** The receiver operating characteristic analysis results for texture parameters associated with the lymphomatous transformation of the parotid gland’s parenchyma in patients with primary Sjögren’s syndrome.

Parameter	Cutoff	AUC	Se (%)	Sp (%)	+LR	−LR	Youden Index	*p*
CH4S6SumVarnc	>207.62	0.800	88.24 (63.6–98.5)	64.58 (49.5–77.8)	2.49 (1.64–3.79)	0.18 (0.04–0.68)	0.528	<0.0001
WavEnHL_s-4	>388.84	0.713	58.82 (32.9–81.6)	81.25 (67.4–91.1)	3.14 (1.54–6.39)	0.51 (0.28–0.91)	0.400	0.0021
Perc90	>33,363	0.816	76.47 (50.1–93.2)	79.17 (65.0–89.5)	3.67 (1.99–6.76)	0.30 (0.12–0.71)	0.554	<0.0001
Mean	>33,233.87	0.821	76.47 (50.1–93.2)	81.25 (67.4–91.1)	4.08 (2.14–7.78)	0.29 (0.12–0.69)	0.577	<0.0001
CV4S6InvDfMom	<0.145	0.875	88.24 (63.6–98.5)	77.08 (62.7–88.0)	3.85 (2.23–6.65)	0.15 (0.04–0.57)	0.653	<0.0001
CH3S6Correlat	>0.321	0.746	52.94 (27.8–77.0)	89.58 (77.3–96.5)	5.08 (1.98–13.05)	0.53 (0.31–0.88)	0.425	0.0008
Perc1	>33,006	0.787	88.24 (63.6–98.5)	62.50 (47.4–76.0)	2.35 (1.57–3.53)	0.19 (0.05–0.70)	0.507	<0.0001

The 95% confidence interval values are shown in parentheses. AUC = area under curve; Se = sensitivity; Sp = specificity; +LR = positive likelihood ratio; −NR = negative likelihood ratio; *p* = statistical significance level; SumVarnc = Sum Variance; WavEnHL = Wavelet Energy High-Level; Perc = Percentile; InvDfMom = Inverse difference moment; Correlat = correlation.

**Table 5:** Multivariate analysis results revealing the texture features independently linked to lymphoma development in patients with primary Sjögren’s syndrome.

Independent Variables	Coefficient	Std. Error	*p*	VIF
(Constant)	−27.7065			
CH4S6SumVarnc	0.00417	0.001495	0.0072	3.284
WavEnHL_s-4	0.00009	0.0001514	0.5478	1.303
Perc90	−0.00242	0.0006944	0.001	15.771
Mean	0.00423	0.001474	0.0058	27.424
CV4S6InvDfMom	3.3534	0.8530	0.0002	1.529
CH3S6Correlat	0.0356	0.3662	0.9229	3.776
Perc1	−0.001	0.001147	0.3841	7.217
R^2^	0.5524			
R^2^-adjusted	0.4975			
MCC	0.7433			
RSD	0.3140			

Std. Error = standard error; *p* = statistical significance level; VIF = Variance Inflation Factor; R^2^ = coefficient of determination; R^2^-adjusted = coefficient of determination adjusted for the number of independent variables in the regression model; MCC = multiple correlation coefficient; RDS = residual standard deviation; SumVarnc = Sum Variance; WavEnHL = Wavelet Energy High-Level; Perc = Percentile; InvDfMom = Inverse difference moment; Correlat = correlation.

**Table 6:** The receiver operating characteristic analysis for the radiomic model predictive of the lymphomatous transformation of the parotid gland’s parenchyma in patients with primary Sjögren’s syndrome.

Parameter	Cutoff	AUC	Se (%)	Sp (%)	Youden Index	*p*
Radiomic Model	≥1.556	0.931	94.12 (71.3–99.9)	85.42 (72.2–93.9)	0.795	<0.0001

The 95% confidence interval values are shown in parentheses. AUC = area under curve; Se = sensitivity; Sp = specificity; *p* = statistical significance level.

## Data Availability

The data are available only by request.

## References

[1] Tzioufas A.G., Youinou P., Moutsopoulos H.M., Isenberg D.A., Maddison P., Woo P., Glass D., Breedveld F. (2004). Sjögren’s syndrome. Oxford Textbook of Rheumatology.

[2] Baldini C., Zabotti A., Filipovic N., Vukicevic A., Luciano N., Ferro F., Lorenzon M., De Vita S. (2018). Imaging in primary Sjögren’s syndrome: The ‘obsolete and the new’. Clin. Exp. Rheumatol..

[3] De Vita S., Gandolfo S. (2019). Predicting lymphoma development in patients with Sjögren’s syndrome. Expert Rev. Clin. Immunol..

[4] Skarlis C., Raftopoulou S., Mavragani C.P. (2022). Sjogren’s Syndrome: Recent Updates. J. Clin. Med..

[5] Quartuccio L., Isola M., Baldini C., Priori R., Bartoloni Bocci E., Carubbi F., Maset M., Gregoraci G., Della Mea V., Salvin S. (2014). Biomarkers of lymphoma in Sjögren’s syndrome and evaluation of the lymphoma risk in prelymphomatous conditions: Results of a multicenter study. J. Autoimmun..

[6] Fragkioudaki S., Mavragani C.P., Moutsopoulos H.M. (2016). Predicting the risk for lymphoma development in Sjogren syndrome: An easy tool for clinical use. Medicine.

[7] Milic V., Colic J., Cirkovic A., Stanojlovic S., Damjanov N. (2019). Disease activity and damage in patients with primary Sjogren’s syndrome: Prognostic value of salivary gland ultrasonography. PLoS ONE.

[8] Coiffier G., Martel A., Albert J.D., Lescoat A., Bleuzen A., Perdriger A., De Bandt M., Maillot F. (2021). Ultrasonographic damages of major salivary glands are associated with cryoglobulinemic vasculitis and lymphoma in primary Sjogren’s syndrome: Are the ultrasonographic features of the salivary glands new prognostic markers in Sjogren’s syndrome?. Ann. Rheum. Dis..

[9] Bădărînză M., Serban O., Maghear L., Bocsa C., Micu M., Damian L., Felea I., Fodor D. (2020). Shear wave elastography as a new method to identify parotid lymphoma in primary Sjögren Syndrome patients: An observational study. Rheumatol. Int..

[10] Kato H., Kanematsu M., Goto H., Mizuta K., Aoki M., Kuze B., Hirose Y. (2012). Mucosa-associated lymphoid tissue lymphoma of the salivary glands: MR imaging findings including diffusion-weighted imaging. Eur. J. Radiol..

[11] Jousse-Joulin S., Coiffier G. (2020). Current status of imaging of Sjogren’s syndrome. Best practice & research. Clin. Rheumatol..

[12] Lambin P., Rios-Velazquez E., Leijenaar R., Carvalho S., van Stiphout R.G., Granton P., Zegers C.M., Gillies R., Boellard R., Dekker A. (2012). Radiomics: Extracting more information from medical images using advanced feature analysis. Eur. J. Cancer.

[13] Gillies R.J., Kinahan P.E., Hricak H. (2016). Radiomics: Images Are More than Pictures, They Are Data. Radiology.

[14] Gerlinger M., Rowan A.J., Horswell S., Math M., Larkin J., Endesfelder D., Gronroos E., Martinez P., Matthews N., Stewart A. (2012). Intratumor heterogeneity and branched evolution revealed by multiregion sequencing. N. Engl. J. Med..

[15] Choi E.R., Lee H.Y., Jeong J.Y., Choi Y.L., Kim J., Bae J., Lee K.S., Shim Y.M. (2016). Quantitative image variables reflect the intratumoral pathologic heterogeneity of lung adenocarcinoma. Oncotarget.

[16] Caruso D., Polici M., Zerunian M., Pucciarelli F., Guido G., Polidori T., Landolfi F., Nicolai M., Lucertini E., Tarallo M. (2021). Radiomics in Oncology, Part 1: Technical Principles and Gastrointestinal Application in CT and MRI. Cancers.

[17] Caruso D., Polici M., Zerunian M., Pucciarelli F., Guido G., Polidori T., Landolfi F., Nicolai M., Lucertini E., Tarallo M. (2021). Radiomics in Oncology, Part 2: Thoracic, Genito-Urinary, Breast, Neurological, Hematologic and Musculoskeletal Applications. Cancers.

[18] Aringhieri G., Fanni S.C., Febi M., Colligiani L., Cioni D., Neri E. (2022). The Role of Radiomics in Salivary Gland Imaging: A Systematic Review and Radiomics Quality Assessment. Diagnostics.

[19] Vukicevic A.M., Milic V., Zabotti A., Hocevar A., De Lucia O., Filippou G., Frangi A.F., Tzioufas A., De Vita S., Filipovic N. (2020). Radiomics-Based Assessment of Primary Sjögren’s Syndrome from Salivary Gland Ultrasonography Images. IEEE J. Biomed. Health Inform..

[20] Vukicevic A.M., Radovic M., Zabotti A., Milic V., Hocevar A., Callegher S.Z., De Lucia O., De Vita S., Filipovic N. (2021). Deep learning segmentation of Primary Sjögren’s syndrome affected salivary glands from ultrasonography images. Comput. Biol. Med..

[21] Muntean D.D., Bădărînză M., Ștefan P.A., Lenghel M.L., Rusu G.M., Csutak C., Coroian P.A., Lupean R.A., Fodor D. (2022). The Diagnostic Value of MRI-Based Radiomic Analysis of Lacrimal Glands in Patients with Sjögren’s Syndrome. Int. J. Mol. Sci..

[22] Chu C., Wang F., Zhang H., Zhu Y., Wang C., Chen W., He J., Sun L., Zhou Z. (2018). Whole-volume ADC Histogram and Texture Analyses of Parotid Glands as an Image Biomarker in Evaluating Disease Activity of Primary Sjögren’s Syndrome. Sci. Rep..

[23] van Ginkel M.S., Glaudemans A.W.J.M., van der Vegt B., Mossel E., Kroese F.G.M., Bootsma H., Vissink A. (2020). Imaging in Primary Sjögren’s Syndrome. J. Clin. Med..

[24] Vivino F.B. (2017). Sjogren’s syndrome: Clinical aspects. Clin. Immunol..

[25] Tonami H., Matoba M., Kuginuki Y., Yokota H., Higashi K., Yamamoto I., Sugai S. (2003). Clinical and imaging findings of lymphoma in patients with Sjögren syndrome. J. Comput. Assist. Tomogr..

[26] Shiboski C.H., Shiboski S.C., Seror R., Criswell L.A., Labetoulle M., Lietman T.M., Rasmussen A., Scofield H., Vitali C., Bowman S.J. (2017). 2016 American College of Rheumatology/European League Against Rheumatism Classification Criteria for Primary Sjögren’s Syndrome: A Consensus and Data-Driven Methodology Involving Three International Patient Cohorts. Arthritis Rheumatol..

[27] Seror R., Bowman S.J., Brito-Zeron P., Theander E., Bootsma H., Tzioufas A., Gottenberg J.E., Ramos-Casals M., Dörner T., Ravaud P. (2015). EULAR Sjögren’s syndrome disease activity index (ESSDAI): A user guide. RMD Open.

[28] Michal Strzelecki M., Szczypinski P., Materka A., Klepaczko A. (2013). A software tool for automatic classification and segmentation of 2D/3D medical images. Nuclear Instruments and Methods in Physics Research Section A: Accelerators, Spectrometers, Detectors and Associated Equipment.

[29] Mărginean L., Ștefan P.A., Lebovici A., Opincariu I., Csutak C., Lupean R.A., Coroian P.A., Suciu B.A. (2022). CT in the Differentiation of Gliomas from Brain Metastases: The Radiomics Analysis of the Peritumoral Zone. Brain Sci..

[30] Ardakani A.A., Rasekhi A., Mohammadi A., Motevalian E., Najafabad B.K. (2018). Differentiation between metastatic and tumour-free cervical lymph nodes in patients with papillary thyroid carcinoma by grey-scale sonographic texture analysis. Pol. J. Radiol..

[31] Csutak C., Ștefan P.-A., Lenghel L.M., Moroșanu C.O., Lupean R.-A., Șimonca L., Mihu C.M., Lebovici A. (2020). Differentiating High-Grade Gliomas from Brain Metastases at Magnetic Resonance: The Role of Texture Analysis of the Peritumoral Zone. Brain Sci..

[32] Lupean R.A., Ștefan P.A., Feier D.S., Csutak C., Ganeshan B., Lebovici A., Petresc B., Mihu C.M. (2020). Radiomic Analysis of MRI Images is Instrumental to the Stratification of Ovarian Cysts. J. Pers. Med..

[33] De Vita S., Gandolfo S., Zandonella Callegher S., Zabotti A., Quartuccio L. (2018). The evaluation of disease activity in Sjögren’s syndrome based on the degree of MALT involvement: Glandular swelling and cryoglobulinaemia compared to ESSDAI in a cohort study. Clin. Exp. Rheumatol..

[34] Takagi Y., Sumi M., Sumi T., Ichikawa Y., Nakamura T. (2005). MR microscopy of the parotid glands in patients with Sjogren’s syndrome: Quantitative MR diagnostic criteria. AJNR Am. J. Neuroradiol..

[35] Kojima I., Sakamoto M., Iikubo M., Kumamoto H., Muroi A., Sugawara Y., Satoh-Kuriwada S., Sasano T. (2017). Diagnostic performance of MR imaging of three major salivary glands for Sjögren’s syndrome. Oral Dis..

[36] Niemelä R.K., Pääkkö E., Suramo I., Takalo R., Hakala M. (2001). Magnetic resonance imaging and magnetic resonance sialography of parotid glands in primary Sjogren’s syndrome. Arthritis Rheum..

[37] Zhu L., Zhang C., Hua Y., Yang J., Yu Q., Tao X., Zheng J. (2016). Dynamic contrast-enhanced MR in the diagnosis of lympho-associated benign and malignant lesions in the parotid gland. Dento Maxillo Facial Radiol..

[38] Stoia S., Băciuț G., Lenghel M., Badea R., Csutak C., Rusu G.M., Băciuț M., Tamaș T., Boțan E., Armencea G. (2021). Cross-sectional imaging and cytologic investigations in the preoperative diagnosis of parotid gland tumors–An updated literature review. Bosn. J. Basic Med. Sci..

[39] Izumi M., Eguchi K., Nakamura H., Nagataki S., Nakamura T. (1997). Premature fat deposition in the salivary glands associated with Sjögren syndrome: MR and CT evidence. Am. J. Neuroradiol..

[40] Gadodia A., Bhalla A.S., Sharma R., Thakar A., Parshad R. (2011). Bilateral parotid swelling: A radiological review. Dentomaxillofacial Radiol..

[41] Nakatsu M., Hatabu H., Itoh H., Morikawa K., Miki Y., Kasagi K., Shimono T., Shoji K., Shimada Y., Imamura M. (2000). Comparison of short inversion time inversion recovery (STIR) and fat-saturated (chemsat) techniques for background fat intensity suppression in cervical and thoracic MR imaging. J. Magn. Reson. Imaging.

[42] Shimamoto H., Tsujimoto T., Kakimoto N., Majima M., Iwamoto Y., Senda Y., Murakami S. (2018). Effectiveness of the periodically rotated overlapping parallel lines with enhanced reconstruction (PROPELLER) technique for reducing motion artifacts caused by mandibular movements on fat-suppressed T2-weighted magnetic resonance (MR) images. Magn. Reson. Imaging.

[43] Dennie C., Thornhill R., Sethi-Virmani V., Souza C.A., Bayanati H., Gupta A., Maziak D. (2016). Role of quantitative computed tomography texture analysis in the differentiation of primary lung cancer and granulomatous nodules. Quant. Imaging Med. Surg..

[44] van Griethuysen J., Fedorov A., Parmar C., Hosny A., Aucoin N., Narayan V., Beets-Tan R., Fillion-Robin J.C., Pieper S., Aerts H. (2017). Computational Radiomics System to Decode the Radiographic Phenotype. Cancer Res..

[45] Clausi D.A. (2002). An analysis of co-occurrence texture statistics as a function of grey level quantization. Can. J. Remote Sens..

[46] Unser M. (1986). Sum and difference histograms for texture classification. IEEE Trans. Pattern Anal. Mach. Intell..

[47] Haralick R.M., Shanmugam K., Dinstein I. (1973). Textural Features for Image Classification. IEEE Trans. Syst. Man Cybern..

[48] Collins G.S., Reitsma J.B., Altman D.G., Moons K.G. (2015). Transparent Reporting of a multivariable prediction model for Individual Prognosis or Diagnosis (TRIPOD): The TRIPOD statement. Ann. Intern. Med..

[49] van Timmeren J.E., Cester D., Tanadini-Lang S., Alkadhi H., Baessler B. (2020). Radiomics in medical imaging—”How-to” guide and critical reflection. Insights Into Imaging.

[50] Shur J.D., Doran S.J., Kumar S., Ap Dafydd D., Downey K., O’Connor J.P.B., Papanikolaou N., Messiou C., Koh D.M., Orton M.R. (2021). Radiomics in Oncology: A Practical Guide. Radiographics.

[51] Qin B., Wang J., Yang Z., Yang M., Ma N., Huang F., Zhong R. (2015). Epidemiology of primary Sjögren’s syndrome: A systematic review and meta-analysis. Ann. Rheum. Dis..

[52] Buch K., Kuno H., Qureshi M.M., Li B., Sakai O. (2018). Quantitative variations in texture analysis features dependent on MRI scanning parameters: A phantom model. J. Appl. Clin. Med. Phys..

[53] Baeßler B., Weiss K., Pinto Dos Santos D. (2019). Robustness and Reproducibility of Radiomics in Magnetic Resonance Imaging: A Phantom Study. Investig. Radiol..

[54] Bologna M., Corino V.D.A., Montin E., Messina A., Calareso G., Greco F.G., Sdao S., Mainardi L.T. (2018). Assessment of Stability and Discrimination Capacity of Radiomic Features on Apparent Diffusion Coefficient Images. J. Digit. Imaging.

[55] Cattell R., Chen S., Huang C. (2019). Robustness of radiomic features in magnetic resonance imaging: Review and a phantom study. Vis. Comput. Ind. Biomed. Art.

[56] Peerlings J., Woodruff H.C., Winfield J.M., Ibrahim A., Van Beers B.E., Heerschap A., Jackson A., Wildberger J.E., Mottaghy F.M., DeSouza N.M. (2019). Stability of radiomics features in apparent diffusion coefficient maps from a multi-centre test-retest trial. Sci. Rep..

[57] Um H., Tixier F., Bermudez D., Deasy J.O., Young R.J., Veeraraghavan H. (2019). Impact of image preprocessing on the scanner dependence of multi-parametric MRI radiomic features and covariate shift in multi-institutional glioblastoma datasets. Phys. Med. Biol..

[58] Jethanandani A., Lin T.A., Volpe S., Elhalawani H., Mohamed A.S.R., Yang P., Fuller C.D. (2018). Exploring Applications of Radiomics in Magnetic Resonance Imaging of Head and Neck Cancer: A Systematic Review. Front. Oncol..

[59] Lambin P., Leijenaar R.T.H., Deist T.M., Peerlings J., de Jong E.E.C., van Timmeren J., Sanduleanu S., Larue R.T.H.M., Even A.J.G., Jochems A. (2017). Radiomics: The bridge between medical imaging and personalized medicine. Nat. Rev. Clin. Oncol..

[60] Zwanenburg A., Vallières M., Abdalah M.A., Aerts H.J.W.L., Andrearczyk V., Apte A., Ashrafinia S., Bakas S., Beukinga R.J., Boellaard R. (2020). The Image Biomarker Standardization Initiative: Standardized Quantitative Radiomics for High-Throughput Image-based Phenotyping. Radiology.

[61] Xue C., Yuan J., Zhou Y., Wong O.L., Cheung K.Y., Yu S.K. (2022). Acquisition repeatability of MRI radiomics features in the head and neck: A dual-3D-sequence multi-scan study. Vis. Comput. Ind. Biomed. Art.

[62] Bianchini L., Botta F., Origgi D., Rizzo S., Mariani M., Summers P., García-Polo P., Cremonesi M., Lascialfari A. (2020). PETER PHAN: An MRI phantom for the optimisation of radiomic studies of the female pelvis. Phys. Med..

